# The role of a cosmetologist in the area of health promotion and health education: A systematic review

**DOI:** 10.34172/hpp.2020.52

**Published:** 2020-11-07

**Authors:** Monika Michalak

**Affiliations:** Institute of Medical Sciences, Collegium Medicum, Jan Kochanowski University, Kielce, Poland

**Keywords:** Beauty salons, Health education, Health promotion, Prevention, Women

## Abstract

**Background:** Contemporary cosmetology, apart from beautifying and caring for the human body, deals also with prevention aimed at maintaining health and physical fitness as long as possible. The profession of a cosmetologist so understood is closely related to the modern concept of health promotion, the part of which is health education. The objective of this review was to evaluate whether a cosmetologist may be a health promoter, and whether a beauty salon mayserve as a place for conducting educational programs.

**Methods:** A systematic review was done using several electronic databases such as PubMed(including MEDLINE), Web of Science Core Collection, Scopus, Embase, and Academic Search Ultimate (EBSCO) and related keywords. The studies published in English between 2008 and 2018 which had specifically mentioned the role of a cosmetologist in the area of health promotion and health education were included.

**Results:** In total, 7 articles met the study criteria. It was found that cosmetologists have the potential to promote pro-health activities. The results of this review also suggest that beauty salons are suitable places for increasing pro-health awareness and can be successfully used to conduct educational programs about healthy lifestyle, as well as skin, breast and cervical cancer prevention.

**Conclusion:** A well-educated and aware of health risks cosmetologist seems to be the right person to transmit and spread knowledge about the proper lifestyle in her workplace and the local environment. A beauty salon, as a place of social interaction, may constitute an area of implementation of pro-health educational programs.

## Introduction

The development of ideas concerning health has a long history. The interest in medicinal raw materials was evolving in many regions of the world, including Egypt, China or India. A lot of information about raw materials used in medicine and daily hygiene can be found in the form of direct sources such as Ebers papyrus, containing ready recipes that facilitate recovery. The civilization which put much emphasis on a rational way of life, with provisions on hygiene related to nutrition and gymnastics, was undoubtedly ancient Egypt. The Egyptians were the first to discover the healing and antiseptic properties of aromatic oils and spices. There is also much information about beauty treatments and products coming from ancient times.^1,2^ The field of cosmetics, taking the advantage of the progress of civilization and the development of science, turned over the years into a scientific-practical field called cosmetology.^3^ Contemporary cosmetology aims at not only care and increasing the attractiveness of the human body, but also the promotion of a healthy lifestyle, regarding well-balanced diet, proper physical activity and awareness of the external environment threats as well as shaping pro-health behaviors. So understood cosmetology is closely related to the modern vision of health promotion, the part of which is health education.^4,5^ The literature review shows that cosmetologists undertake talks with their patients on the topics related to health and illness.^6,7^ Moreover beauty salons are indicated by many authors as suitable places for increasing the awareness and educating patients about positive health behaviors^7-13^ (Table 1).

The academic literature review shows that beauty salons are indicated by many authors as suitable places for increasing the awareness and educating clients about skin,^14^ breast^12,13^ and cervical^7^ cancer prevention, diabetes,^15^stroke,^10^ menopause,^11^ as well as for promoting vaccination against human papilloma virus (HPV),^16^ screening colonoscopy^17^ and life-saving falls prevention.^18^ The results of many studies also suggest that beauty salons can be successfully employed to distribute health-related information.^7,19^

The literature data indicate the role of a cosmetologist in health promotion and health education. However, in recent years no systematic review has been done discussing health education and promotion programs implemented in beauty salons involving cosmetologists. Taking this context into account, the objective of this paper is to discuss whether cosmetologists may act as potential health educators and whether pro-health activities conducted by cosmetologists contribute to raising the customers’ awareness about healthy lifestyle and motivate them to undertake healthy behaviors.

## Materials and Methods

Literature was reviewed to find interventions for educational and health promoting programs implemented in beauty salons involving cosmetologists. Studies were searched for using electronic databases: PubMed (including MEDLINE), Web of Science Core Collection, Scopus, Embase and Academic Search Ultimate (EBSCO).^20^ The following keywords, as well as various keyword combinations such as: health education, health promotion, beauty salon and cosmetologist were used in the search. The search started with one keyword, and additional keywords were added to reduce the number of records. The research was conducted with the aid of an experienced librarian.

The selected studies met the following criteria: (1) they were published between the period of 2008–2018, (2) they were written in English, (3) they were full-text original research published in academic peer-reviewed journal, and (4) they described the actions concerning health promotion and education conducted in beauty salons involving cosmetologists. Once the articles were selected on the basis of the inclusion criteria, they were evaluated on basis of the exclusion criteria. The articles were excluded from this review if: (1) they were identified as a review, conference abstract, meeting, letter, note, or editorial, (2) they were written in languages other than English, and (3) they concerned health promotion and education carried out by such professionals as hairdressers or stylists.

**Figure 1:**
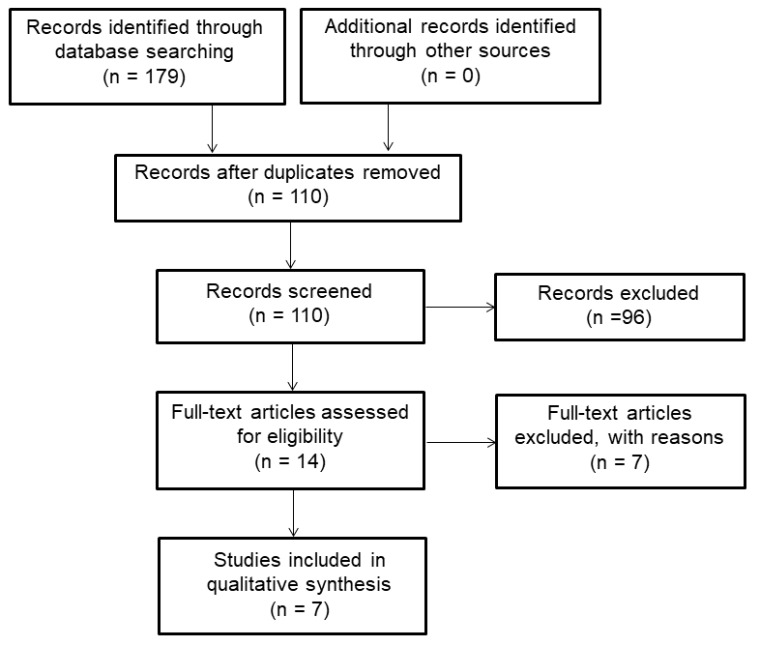
Flow chart of the articles selection process.

After eliminating duplicate studies, titles and abstracts of all relevant articles were screened initially to see if they met the inclusion criteria. The full-text articles were then read and reviewed on the basis of the predefined eligibility criteria. The search was confined to the published articles only and the authors were not contacted for any clarifications. No attempt was made to assess gray literature. Figure 1 presents the findings of literature search. Preferred Reporting Items for Systematic Reviews and Meta-Analyses (PRISMA) guidelines were applied for processing the steps of study.^21^

The extracted data are presented in the form of a table in this review. This systematic review was conducted to collect and summarize the research concerning educational and health promoting programs implemented in beauty salons involving cosmetologists.

## Results

The primary search provided 179 published articles, of which 69 articles were eliminated after removal of duplicates. Ninety-six publications were excluded from the study due to previously defined exclusion criteria. The data extraction method yielded a total of 7 studies evaluating distinct interventions that met the inclusion criteria and were analysed in this systematic review (Figure 1).^6-8,10,14,22,23^Table 2 presents a summary of important interventions with regard to health promotion and pro-health educational programs conducted in beauty salons involving cosmetologists. The studies included in this systematic review were summarized on the basis of samples, methods, assessment of intervention effectiveness, and key findings.

With regard to the content of the 7 articles, 4 of them were focused on cancer prevention, and the other three concerned healthy lifestyle and general health. All of the discussed interventions conducted in the beauty salons concerned women and involved cosmetologists. In all of them the cosmetologists were surveyed in terms of comfort and interest in promoting health topics or took part in the programs implemented in beauty salons (including serving as health promoter, recruiting study participants, administering surveys, distributing training components of the program (materials, brochures, pamphlets, information packets etc.), as well as educating clients or implementing the motivational sessions during appointments).

It was found that cosmetologists have the potential to operate as community health workers to promote pro-health activities and assist with increasing population-based pro-health awareness. The results of this studies also suggest that beauty salons constitute an appropriate setting for promoting a healthy lifestyle (physical activity and healthy eating)^8^ and educating clients about skin,^14,22^ breast^23^ and cervical^7^ cancer prevention, as well as stroke.^10^ The results of six,^7,8,10,14,22,23^ out of seven discussed research studies indicate that cosmetologists are interested in the conducted educational programs and most of them are either comfortable or very comfortable promoting health topics among their clients.

As the results of the discussed interventions showed, educational and health promoting programs conducted with the aid of cosmetologists raised the customers’ awareness about healthy lifestyle and motivated them to undertake healthy behaviors. A skin cancer prevention and early detection program^14^ increased cosmetologists’ awareness of skin cancers as well as their comfort with talking to their clients about skin cancer prevention (from 55% to 100%) and skin cancer early detection (from 64% to 100%), as well as referring a client to a health care provider (from 73% to 100%). An intervention aimed to increase the awareness of the relation between tanning bed use and skin cancer^22^ showed that the cosmetologists talked more often with their clients about cancer risk connected with tanning bed use, as well as less frequently offered tanning beds to their clients. The results of a healthy lifestyle promotion program^8^ indicated that the intervention significantly influenced the increase in daily fruit and vegetable consumption as well as the mean intake of water in the treatment group. Health education program on stroke^10^ showed that the knowledge of at least three warning stroke signs increased among the beauty salon clients (from 40.7% to 50.6%) at a five-month follow-up.

## Discussion

Given the subject matter of this article, it is reasonable to refer to the definition of health promotion, referred to as „a process that enables people to control their own health and improve it by making pro-health choices and decisions, shaping needs and competences to solve health problems as well as increasing health potential”.^24^ An inherent element of health promotion is health education aimed at providing information and knowledge about health and illness. Health education, focused on changing harmful behaviors, heads to encourage individuals to modify their life-style into a healthy one. Health education, in accordance with the broadly understood prophylaxis, should be offered not only to the ill, but most of all to healthy people.^5^

A cosmetologist can conduct preventive activities regarding care and delaying signs of skin aging, as well as encourage and initiate changes in health attitudes and behaviors, for example through an individual conversation, which gives a great opportunity for educational impact, as it concerns current health situation of a particular person. Scientific works confirm the common view that beauty and health are related.^6^ Solomon et alreported that approximately 18% of the talk that occurs in a salon is health related.^25^ By contrast, the results of the research conducted by Lee et al indicate that 78% of the cosmetologists talked about health-related issues with their customers and 61% of the customers reported discussing health-related issues with their cosmetologists.^7^ The academic literature review shows that cosmetologists undertake talks with their patients on the topics related to diet, physical activity, weight control, sun exposure, aging health, stress management in everyday life, smoking, illness (e.g., cancer, diabetes, depression, high blood pressure), medical care, routine health check-ups, mammography, self-care, as well as reproductive health (e.g., menstrual cycles, perineal hygiene, menopause, and sexuality), pregnancy, motherhood and child health.^6,7,9,19,25^ The literature data confirm that due to the possibility of reaching a wide audience, at the workplace perceived as a place of relaxation as well as favouring openness and creating conditions for building a relationship of trust, a cosmetologist may be optimal as health promoter.^4,9,19,25,26^ Furthermore, most women visit cosmetology salon regularly, tend to return to the same cosmetologist over a period of years and spend approximately 1.5 to 3 hours or more with them.^7,25^

In the light of the above, the cosmetologists, perceived as ‘‘natural helpers’’ thanks to providing their clients with health information and as trusted members of the community, gain new tasks and opportunities to improve and promote health in their workplace and local environment.^4,23,25^

### Cosmetologist as a skin health promoter 

A cosmetologist seems to be a good health promoter, especially a skin health promoter. The scope of professional duties of a cosmetologist may include the activities supporting the prevention of skin cancer, including education of the society concerning the harmfulness of ultraviolet radiation both natural (solar radiation) and artificial (solaria) as well as methods of broadly understood sun protection.^27^

Overexposure to ultraviolet (UV) radiation from the sun and artificial sources, which plays an important role in the development of skin cancer, is of considerable public health concern.^28-30^ Health promotion and effective popularization of knowledge about the negative health effects of exposure to ultraviolet radiation can significantly contribute to reducing the incidence of skin cancer, including basal cell carcinoma, squamous cell carcinoma and melanoma, for which the etiological factor is UV radiation.^31-33^ It is important to increase patients’ awareness of the risk factors for malignant skin tumor. Apart from intense sunlight exposures leading to skin burns, especially those in young age and chronic exposure to UV,^34-36^ risk factors include also phenotypical sensitivity to the sun, the organism’s decreased immunity, the past cancers of skin, occurrence of neoplasms in the family, genetically determined diseases (*xeroderma pigmentosum* ), PUVA treatment as well as a high quantity or constant irritation and injuries of pigmented nevi.^37-40^ The cosmetologist should pay attention to the above risk factors at the diagnosis stage and suggest dermatological consultations when they occur.

Educating society about the methods of sunscreen protection is, apart from showing UV harmfulness, the best strategy to reduce the incidence of skin cancer and the determinant of its health.^27,41^ An important element of sun protection is appropriate behavior during sunlight exposure, minimizing the amount of time spent in the sun, especially between 10 AM and 5 PM, use of protective clothing and sunscreen preparations.^28,41,42^ The cream with sun filter should provide protection against UVA and UVB radiation, be waterproof, non-toxic and do not cause irritation and allergic reaction. The application of UV filters with a wide-range of protection not only prevents cancer, but also provides protection against sunburn, and allows for increasing the planned exposure time in the sun.^41^ The cosmetologist should inform and make patients aware that preparations protecting against UV radiation should be used throughout the year, especially for the UV exposures longer than 20 minutes. A sufficiently thick layer (2 mg/cm^2^) of a sun cream should be applied every 2-3 hours, thoroughly on the whole body, 20-30 minutes before going outside.^43^ The cosmetologist can also help in choosing a cream of appropriate SPF (sun protection factor), depending on the skin phototype.^44,45^ For example, individuals with skin phototype I should choose sunscreen preparation with the highest degree of SPF protection (SPF 50+), those with skin phototype II – at least SPF 30, while those with darker skin phototypes - SPF 15 preparation. In their daily work, cosmetologists should also make patients aware that the best form of skin cancer and photoaging prevention is taking extensive action. Therefore, it is pointed out that besides preparations used externally, oral and systemic photoprotection plays an important role in protection against harmful UV radiation. It has been proven that natural antioxidants such as carotenoids, vitamins C, E and plant extracts rich in polyphenols, by their antiradical activity, effectively complement protection against UV radiation.^41^ A different problem in the prevention of skin cancers are artificial sources of UV radiation. The results of epidemiological studies indicate that there is a link between the incidence of melanoma, as well as squamous and basal cell carcinomas and the use of sunbeds.^46-48^ It was shown that exposure to UVA and UVB radiation from indoor tanning devices cause DNA damage in skin cells. While UVB is associated with direct DNA damage, UVA exposure is associated with indirect DNA damage through the production of reactive oxygen species.^49-51^ Since treatments offered by a cosmetologist should be primarily harmless to health, solarium services should be excluded from cosmetological offer. Taking into account patient’s health, the cosmetologist should also promote the untanned skin of natural colour.^27^

The cosmetologists should also pay attention to the presence of alarming skin lesions on their patients’ body and, in justified cases, suggest consulting an appropriate medical specialist.^52^ The cosmetologists ought to be aware that in their daily work they may contribute to a decrease in mortality rates due to skin cancers. A proper interview carried out before the treatment, vigilance in evaluating any skin lesions and referring patients with worrying symptoms to dermatologists or oncologic surgeons may speed up the process of making the correct diagnosis of cancerous changes and thus the initiation of treatment in the early stage of the disease.^27^ Dorman et al as part of the program entitled *Talkin ’ About Better Skin* (TABS) conducted questionnaire surveys assessing the readiness of cosmetologists to talk about health with their clients.^14^ The aim of the study was also to estimate the level of the cosmetologists’ knowledge, motivation and skills to disseminate information concerning prevention and early detection of skin cancer after participation in a 2-hour educational program. The results of the survey conducted among 189 cosmetologists after five months of the implementation of the pro-health program showed that 89% of the cosmetologists talked with their clients on the topics related to skin health, including sunscreen protection. What is more, 63% of the cosmetologists knew how to tell if a skin area needed to be examined by a doctor for possible cancer and 49% of them had successfully referred a patient for the examination of suspicious skin lesions. The results of the intervention carried out by Ng et al showed that cosmetologists appear to be a group of healthcare professionals who can contribute to increase skin cancer awareness in population^22^. The role of a cosmetologist in educating patients in the area of skin disease prevention is also emphasized by Antoszewski et al.^52^ The results of the conducted research show that being aware of the skin cancer risk, the cosmetologists observe skin changes on the patient’s body during treatments and suggest a consultation with a specialist.^52^

The literature review indicates that beauty salons may also be suitable places for increasing the awareness about pregnancy, motherhood and child health.^6,9,19,25^ One of the studies showed that pregnancy and motherhood were the most frequently discussed health-related topics in beauty salons.^25^ However, the research done by Ahlers-Schmidt et al demonstrated that only about one-third of the cosmetologists taking part in the study conversed with their clients regarding maternal/child health issues.^6^ This intervention, assessing the role of the cosmetologists as health promoters in the prevention of infant mortality, showed their limited interest in promoting safe sleep behaviors, as the cosmetologists were either unsure or did not feel that infant mortality was a problem in their community. However, as the authors observe, cosmetologists may be more willing to take part in the obesity-prevention programs to reduce infant mortality rate rather than in the safe sleep promotion programs.^6^

### The role of cosmetologist in health education programs

On the basis of the literature data, different ways of the educational programs’ implementation in the beauty salons can be distinguished, e.g. by the independent cosmetologists using their own competences, by the cosmetologists as purposely trained health promoters implementing educational programs developed by external sources, as well as educational activities undertaken in a cosmetology salon with the participation of non-cosmetologist volunteers of pro-health organizations.^8-10,12^

The literature review shows that the cosmetologist may participate in educational programs promoting general health, healthy eating and physical activity, as well as aimed at cancer prevention.

As the literature data show, the cosmetologists’ knowledge and competences justify their inclusion in the breast cancer prevention program.^12^ While performing various care treatments, also in the area of breasts of women in a very wide age range, a cosmetologist can often notice alarming changes in their structure or skin appearance (including lump or mass in the armpit, a change in the size or shape of the breast, change in the color or feel of the skin of the breast, nipple or areola, change in the appearance or sensation of the nipple).^53,54^Furthermore, a cosmetology salon can be a place for disseminating knowledge, breaking the taboo and starting a dialogue about breast cancer.^54^ This is confirmed by the described in the literature educational programs, in which previously trained cosmetologists participated, and which were devoted to breast cancer prevention and carried out on a group of clients of cosmetology salons who, after getting information (through conversation, information brochures), were more inclined to do a breast self-examination and perform mammography.^6,12,23^ Moreover, the patients participating in the educational programs definitely supported the role of a cosmetologist as a health educator.^23^ The results of the research carried out by Asuquo and Olajide showed that breast cancer awareness significantly affect individual’s knowledge of the symptoms and risk factors of breast cancer, as well as sensitizes to prevention and helps shaping health attitudes (e.g. practice of breast self-examination).^53^ Hence, health education has a positive significant role in the reduction of breast cancer, the most common malignant tumor among women worldwide.^53,55^

Lee et al have utilized beauty salons to implement health education programs aimed at adherence to cervical cancer screening.^7^ The results of the research in which 62 participants took part (cosmetologists and customers at the age of 18 or older; the average age was 42.9 years) suggest that cosmetologists can be utilized as potential health educators to deliver cervical cancer-related information to their customers. These findings also suggest that while developing educational programs it is crucial to ensure that cosmetologists are properly trained in the subject matter, which could increase their confidence in their role as health educators.^7^ Other studies indicate that beauty salon may be a place of educating women about cervical cancer, HPV, and vaccination.^16^ The results of the research involving stylists showed that the knowledge of the clients and their attitudes towards HPV vaccination were positively changed (from 33% to 75%). An intention to talk to health care professionals about the vaccine, as well as a willingness to vaccinate against HPV significantly increased after the intervention.^16^ It is worth noting that both the above described and other educational programs are urgently needed to spread knowledge about prevention and early detection of cervical cancer.^56^ These activities are extremely important, because as Wychowaniec et al notice women’s health behaviors regarding cervical cancer prevention are increasingly positive, which is associated with widespread health education.^57^

Johnson et al emphasize that a cosmetologist’s workplace can be used to implement educational programs aimed at promoting healthy diet and physical activity.^8^ In their study, cosmetologists were trained by the research team to disseminate information and conduct motivational sessions encouraging to healthy behaviors. For example, the benefits of fruit and vegetables consumption in relation to chronic diseases were indicated, such as reduction of coronary disease or the influence of potassium on the maintenance of normal blood pressure. What were also highlighted were the advantages of physical activity in relation to chronic diseases, cholesterol and energy levels’ control as well as positive influence of drinking water on digestion, removal of waste products from the body and weight loss. The progress of the project and the course of each training session was consulted with the research team on a weekly basis. It was shown that in the group of women aged 18-70, educated and motivated by the cosmetologists for six weeks to maintain appropriate health behaviors, the consumption of fruit and vegetables increased on average from 1.8 to 3.5 portions per day. Moreover, a trend in increase in water consumption was observed. With regard to physical activity, no significant improvement was observed, due to the difficulty of changing so many behaviours in such a short period of time.^8^ The results of studies by other authors confirm that the topics related to healthy eating are often raised by cosmetologists in the conversations with their clients.^19^ Numerous scientific works provide more and more data highlighting, for example, the protective role of antioxidants, of which such biologically active compounds as resveratrol, curcumin, genistein and epigallocatechin gallate are extensively studied.^58-61^ What also plays an important role in combating radicals, which can cause extensive damage to cellular structures, are carotenoids, including β-carotene, lycopene or astaxanthin. It is extremely important to include these ingredients in the daily diet, as they not only favor health and well-being, but also positively affect the proper functioning of skin.^62-64^ As evidenced by scientific research, the frequent ingredients of nutricosmetics such as carotenoids, vitamins (including E, C and B-group vitamins), coenzyme Q10, micronutrients (including silicon, zinc and selenium), essential unsaturated fatty acids omega-3 and omega-6 (including α-linolenic acid, linoleic acid, γ-linolenic acid) have a beneficial effect on the skin condition.^64-66^ The components of nutricosmetics play a special role in making up the deficiency of ingredients that protect the skin against dehydration and loss of elasticity as well as oxidative damage caused by the exposure to ultraviolet radiation or inflammation, among others.^64^ The nutricosmetics, which scope has changed with the advancement of knowledge in cosmetology and human nutrition, is now understood not only as the art of beautifying, but also as pro-health prophylaxis.^3^

### Implications for future studies 

With regard to implications, there are future directions to be considered as a result of this review. Available scientific literature indicates that health education and promotion programs conducted by cosmetologists contribute to raising the customers’ awareness about healthy lifestyle and motivate them to undertake healthy behaviors, in particular physical activity, proper eating habits and everyday life hygiene. Thus, popularizing the number of well thought-out training programs involving cosmetologists, might be a direction the field needs to take. Furthermore, it is advisable to consider any possibilities of increasing cosmetologists’ involvement in educational programs, especially building their reputation as community health leaders. Cosmetologists may be optimal as health promoters because they see themselves as information resources for their clients^6^. While developing another educational programs, it is worth considering the activities enhancing cosmetologists’ confidence in their roles as lay health educators, as well as maximizing the authenticity of information exchanged between cosmetologists and customers.^7^ It should be also emphasized that the implementation of educational programs requires from the cosmetologists knowledge and the ability to use and adapt modern concepts, methods and educational techniques to the needs of the beauty salons.^4^ Hence, an important future direction for the field is to engage cosmetologists in educational programs which give them an opportunity to grow in knowledge which, in turn, they can make use of in various pro-health activities. The analyzed studies indicated that cosmetologists are interested in receiving more information about health-related topics that can be shared with their clients during appointments. The topics of their interest include healthy eating, diet and weight control, physical activity, aging health, as well as stress management.^6^Among the preferred methods of learning about the subject, cosmetologists ranked watching videos the highest (31.3%), followed by one-on-one talks (25%)^7^. Customers, in turn, indicated one-on-one talks (23.1%) and watching videos (20.5%) as the most preferred learning method.^7^ Moreover, the data concerning educational programs implemented in beauty salons show that the methods the cosmetologists most preferred for sharing health-related information with their clients include the distribution of educational brochures (69.4%), direct conversation with the client (61.2%), placing posters/mirror stickers in the salons (59.2%), as well as recommending consultations with healthcare professionals (44.9%).^9,19^ It shows that this way of conducting health education may be useful in prospective interventions.

The research findings support the view that the cosmetologists equipped with appropriate knowledge are recommended as community health educators and are likely to influence their customers’ health outcomes.^7^

The current review had several limitations and strengths that are worth consideration. The aim of this study was to present the use of a beauty salon as a place to conduct education programs. There are many publications showing the participation of such professionals as hairdressers or stylists in educational and health promoting programs,^16,67,68^ but there is limited information about the role of the cosmetologists in the scientific articles. A strength is that it is a systematic review of the articles published in recent years that highlights the involvement of cosmetologists in health promotion and health education programs.

This paper focuses on the role of a cosmetologist and attempts to address the question whether pro-health activities conducted in beauty salons by cosmetologists contribute to raising the customers’ awareness about healthy lifestyle and motivate them to undertake healthy behaviors. This review showed that while the range of health topics discussed by cosmetologists in beauty salons appears to be quite similar to these talked about by other professionals working in beauty salons, most published evaluations of programs involving cosmetologists focused on such health topics as: healthy lifestyle and general health, as well as cancer prevention. Although these results are encouraging, this review has showed that more research and evaluation of future beauty salon-based interventions involving cosmetologists are needed. Moreover, due to the fact that this review concerns different educational and health promoting programs based on various types of research and sample sizes, certain difficulties in the comparison of the discussed interventions have been observed.

While this systematic review is informative in identifying various dimensions of the design and implementation of health promotion and education programs in beauty salons, it could be less useful for detailed explanations of how and why programs are thought to be effective.

Moreover, all of the discussed studies were conducted in the United States; therefore, it might be difficult to generalize the results of the analysis to other countries. In addition, not all reviewed studies included the characteristics of the cosmetologists involved in the interventions while the scope of competences and the terms of reference of the cosmetologists may be different in individual countries. Moreover, the search results have been evaluated by one author, which may cause a potential bias. Finally, the limitation of this review may also be the inclusion of the articles written only in English language and not expanding the scope of the review to other literature sources, including dissertations, conference articles, and other available data that might broaden our knowledge regarding the role of a cosmetologist in the area of health promotion and health education.

Despite the limitations, this systematic review presents various pro-health activities carried out in beauty salons, as well as sets the direction for new interventions which may be conducted with the involvement of cosmetologists at their workplace. Also, the methods of conducting the interventions preferred by both the cosmetologists and their clients have been identified and may be used in the prospective educational programs.

## Conclusion

Health promotion and health education are important forms of pro-health activities that are and should be increasingly implemented by healthcare professionals within their competences. The current experience shows that a cosmetologist prepared for the role of a health educator, because of his or her knowledge and the possibility to reach a wide range of people, seems to be the right person to share and disseminate information in the area of disease prevention and health promotion. Many authors emphasize that beauty salons can be a suitable place for conducting educational and health promoting activities.

## Acknowledgements

The author is thankful to The Main Library of the Medical University of Silesia in Katowice and its Branch in Sosnowiec for their support.

## Funding

This work was supported under the program of the Minister of Science and Higher Education under the name “Regional Initiative of Excellence” in 2019-2022 project number: 024/RID/2018/19, financing amount: 11.999.000,00 PLN.

## Competing interests

The author declares that she has no competing interests.

## Ethical approval

Not applicable.

## Authors’ contributions

Conception, preparation, editing and manuscript review by the author.

## Disclaimer

The views expressed in this publication are those of the author. The author disclaims any responsibility for any damage incurred as a consequence of the use or application of the content of the article.

**Table 1:** The topics most often discussed between the professionals working in the beauty salons and their clients as well as some examples of educational programs implemented in the beauty salons^4-13^

**Health-related topics discussed with clients**	**Health education programs**
• Diet/weight control • Exercise • Stress management • Healthy aging • Pregnancy/motherhood • Sun exposure • Smoking • Medical care • Chronic illness • Cancer screening	• Health behaviors (fruit and vegetable intake, physical activity, and water consumption) • Menopause • Prevention of infant mortality • Skin cancer prevention • Diabetes education • Stroke • Breast cancer screening • Cervical cancer screening education

**Table 2:** Summary of the reviewed studies

**Educational and health promoting programs**	**Reported intervention group**	**Study setting**	**Way of conducting health education**	**Assessment of intervention effectiveness**	**Findings**	**Study**
Skin cancer prevention and early detection program disseminated through cosmetologists	189 cosmetologists (female, aged 33-44)	Bryan, Texas	Survey assessing current attitudes, skills, and knowledge of skin cancer prevention and early detection among their clients. Topics covered included: sun Safety, the ABCDEs (Asymmetry, Border, Color, Diameter, Evolving) of skin cancer surveillance. Program format consisted of lessons, discussion, and role play.	A survey distributed among cosmetologists over a five-month period	Program results showed improvement in cosmetologists’ awareness of skin cancers (100% of the participants had heard of all three types of skin cancer) . Pre/post data showed that the program increased the number of cosmetologists who were comfortable with talking to clients about skin cancer prevention (from 55% to 100%), talking with clients about skin cancer early detection (from 64% to 100%), and referring a client to a health care provider (from 73% to 100%)	Dorman et al^14^
Educational intervention on tanning beds and melanoma towards skin cancer prevention	185 non-medical skin care professionals (including 95 cosmetologists, 71 estheticians, 19 massage therapists)	Southern California	Survey administered at nine salons or spas assessing knowledge on tanning and skin cancer, a 10-minute oral presentation and educational flyers focusing on: the association between tanning bed use as a risk factor for developing melanoma, understanding of melanoma and its prevention, demonstrating the process of a self skin examination, and encouraging to share the knowledge of the link between tanning beds and melanoma with clients	Pre- and post-intervention survey, with the latter distributed one month later	Survey results showed: more frequent talking with clients about cancer risk connected with tanning bed use, decreased personal tanning bed use, decreased belief that tanning beds are an excellent cosmetic tool	Ng et al^22^
Healthy lifestyle promotion program	20 clients (women, age range: 18–70) of two beauty salons (the study group (n=10), the control group (n=10)	South Carolina	Education of the clients by trained cosmetologists; educational conversation (three scripted motivational sessions), informational packet (on improving diet, physical activity, and water consumption) and a starter kit (some fruit, vegetables and a bottle of water) for the clients.	Pre- and post test questionnaire, after 6 weeks	The study indicated that fruit and vegetable consumption increased significantly after the program for the study group of beauty salon participants in comparison with the control group. The trend towards more water consumption after the test by the study group is encouraging. There were no significant findings concerning physical activity after the program for either group.	Johnson et al^8^
Breast cancer (BC) prevention program	984 clients (women over the age of 20)	San Diego, California	Education of clients by trained cosmetologists; posters, mirror stickers and literature about BC early detection, synthetic breast models to show how BC lumps might feel	Post-intervention phone surveys; after six months	Intervention was well received by the participants and their cosmetologists. Women in the study group reported undergoing mammography significantly more often compared to women in the control group.	Sadler et al^23^
Health education program on cervical cancer screening	62 women (18 cosmetologists and 44 clients, 18 years of age or older)	Albuquerque, New Mexico	The questionnaire assessing participants’ interest in and comfort with discussing, as well as learning about cervical cancer-related information, and delivering cervical cancer-related material	Statistical analysis of the quantitative survey data. The responses obtained from open-ended questions were analyzed using standard, descriptive, and qualitative content analyses	Survey results showed that cosmetologists expressed interest in discussing cervical cancer-related topics (72.2%) and delivering information to their customers (100%). Clients were similarly interested in talking about cervical cancer-related topics (75%) and learning this information from their cosmetologist (88.6%). Both cosmetologists and customers expressed comfort with cervical cancer education in the beauty salon setting (72.2% and 88.6%, respectively)	Lee et al^7^
Health education program on stroke	383 clients (women in the following age groups: 18 to 39 years old (30.3%), 40 to 59 (47.5%), 60 to 75 (19.3%), 75+ (2.9%)	Cincinnati and Atlanta	Education of clients by 30 trained cosmetologists; educational conversation during appointments about risk factors and stroke warning signs (the “FAST” (Face, Arm, Speech, Time) method), educational brochures	De-identified pre- and post-intervention (one after 6 weeks and another after 5 months) surveys that included open-ended questions	The use of a beauty salons as an educational site is a novel approach to stroke education for women. Stroke education in the beauty salons significantly improved knowledge regarding stroke warning signs (after educational intervention, 94% women knew to call 911 for stroke symptoms). Knowledge of stroke risk factors did not improve, however.	Kleindorfer et al^10^
Infant safe sleep promoting program aimed at infant mortality rate reduction disseminated by cosmetologists	149 cosmetologists, out of which 130 are currently working in beauty salons (women between 22 and 84 years)	Sedgwick County	The survey focusing on clientele’s demographics, topics commonly discussed during appointments, comfort and interest in promoting health topics, and the biggest health concerns in their communities.	Statistic analysis of the data obtained from the survey containing 27 questions	Cosmetologists were not highly interested in providing safe sleep education (≤13 %); but also were unsure (56%) or did not feel that infant mortality was a problem (41%) in their community. However, most cosmetologists showed interest in promoting maternal health behaviors (obesity prevention, including exercise and healthy eating) that influence infant mortality	Ahlers-Schmidt et al^6^
